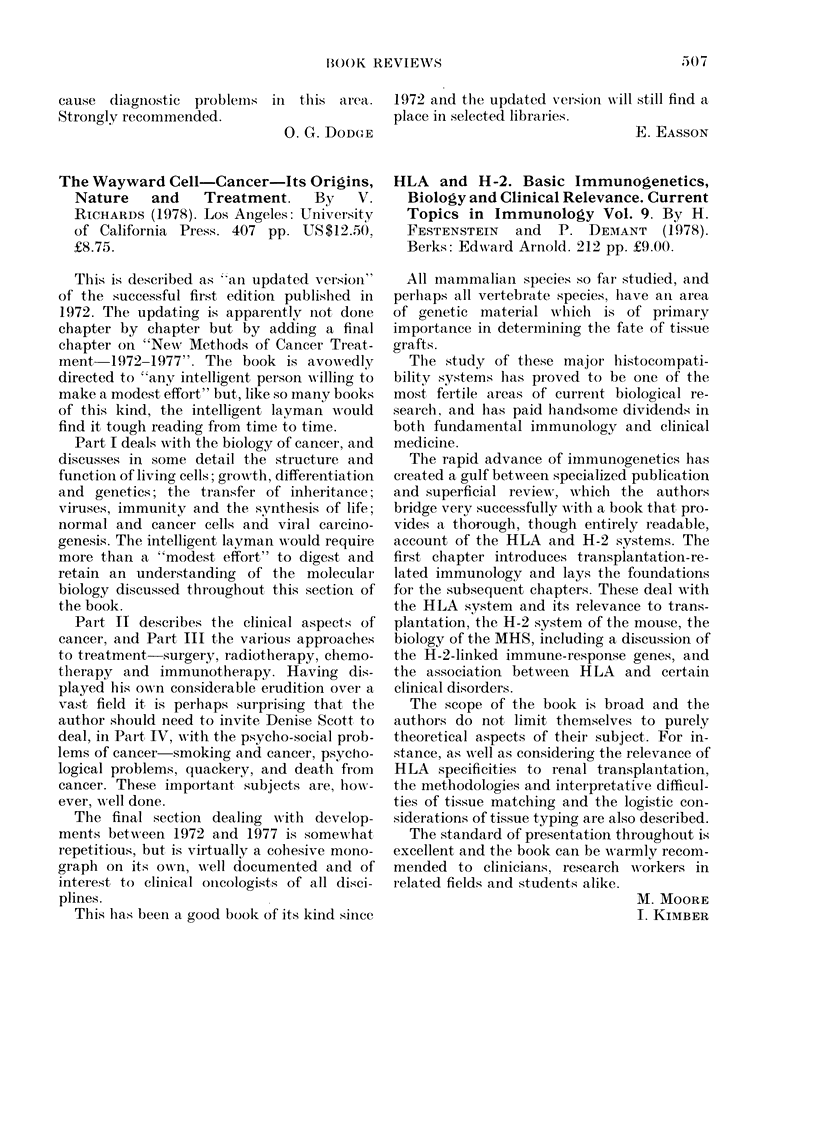# HLA and H-2. Basic Immunogenetics, Biology and Clinical Relevance. Current Topics in Immunology Vol. 9

**Published:** 1979-09

**Authors:** M. Moore, I. Kimber


### HLA and H-2. Basic Immunogenetics, Biology and Clinical Relevance. Current Topics in Immunology Vol. 9. By H. FESTENSTEIN and P. DEMANT (1978). Berks: Edward Arnold. 212 pp. £9.00.

All mammalian species so far studied, and perhaps all vertebrate species, have an area of genetic material which is of primary importance in determining the fate of tissue grafts.

The study of these major histocompatibility systems has proved to be one of the most fertile areas of current biological research, and has paid handsome dividends in both fundamental immunology and clinical medicine.

The rapid advance of immunogenetics has created a gulf between specialized publication and superficial review, which the authors bridge very successfully with a book that provides a thorough, though entirely readable, account of the HLA and H-2 systems. The first chapter introduces transplantation-related immunology and lays the foundations for the subsequent chapters. These deal with the HLA system and its relevance to transplantation, the H-2 system of the mouse, the biology of the MHS, including a discussion of the H-2-linked immune-response genes, and the association between HLA and certain clinical disorders.

The scope of the book is broad and the authors do not limit themselves to purely theoretical aspects of their subject. For instance, as well as considering the relevance of HLA specificities to renal transplantation, the methodologies and interpretative difficulties of tissue matching and the logistic considerations of tissue typing are also described.

The standard of presentation throughout is excellent and the book can be warmly recommended to clinicians, research workers in related fields and students alike.

M. MOORE
I. KIMBER